# Severe accordion effect: Myocardial ischemia due to wire complication during percutaneous coronary intervention: A case report

**DOI:** 10.1186/1757-1626-1-138

**Published:** 2008-09-02

**Authors:** Gerasimos Gavrielatos, Loukas K Pappas, Prodromos Anthopoulos, Anastasios Salachas, Georgios Ifantis, Ioannis Antonellis

**Affiliations:** 1 2nd Department of Cardiology, Evangelismos General Hospital, Athens, Greece; 2 Cardiac Catheterization and Interventional Cardiology Department, Evangelismos General Hospital, Athens, Greece

## Abstract

A mechanical alteration during manoeuvring of stiff guidewires in tortuous coronary arteries frequently induces vessel wall shortening and coronary psedostenosis, referred as accordion phenomenon. Subtraction of the guidewires normally leads to the entire resolution of the lesions. A case of this transient angiographic finding, during percutaneous coronary intervention in a tortuous right coronary artery, which resulted in a flow limiting effect and myocardial ischemia, is described in the present report. Differential diagnosis from potential procedure complications and interventional methodology issues are discussed, while similar reports are reviewed.

## Introduction

Accordion phenomenon is a transient angiographic defect observed during the course of percutaneous coronary intervention (PCI) in tortuous vessels. Herein is described a case of accordion effect in right coronary artery accompanied by ischemic electrocardiographic alterations and severe chest pain. Current interventional practise and technical details are being discussed.

## Case presentation

A 60-year-old female with a history of hypertension and dyslipidemia visited the emergency department due to recurrent episodes of progressive, effort-related angina for the past two months. Physical examination revealed a mild systolic murmur best heart at the apex and her blood pressure was 150/85 mm Hg. Electrocardiogram (ECG) showed T wave inversion in leads I, AVL, V4–V6. The levels of cardiac enzymes were between normal ranges. Two-dimensional echocardiography showed hypokinesis of the inferoposterior and lateral wall with ejection fraction of 50% and mitral regurgitation (grade I/IV). The patient was admitted in hospital and treated with aspirin 325 mg, oral nitrates and clopidogrel 75 mg × 1.

**Figure 1:**
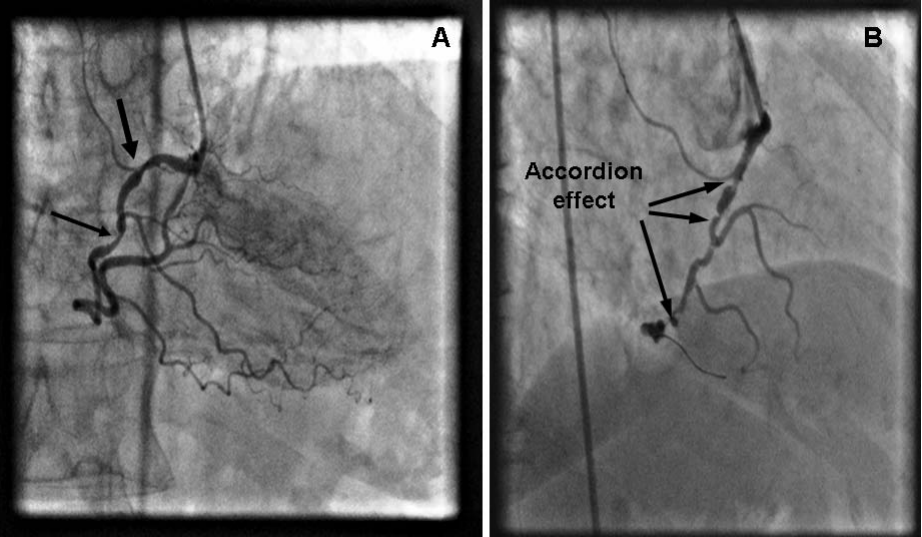
**(a) Coronary angiography of the right coronary artery in the AP-cranial projection demonstrating two stenotic lesions of 70% and 80% (arrows), located at the proximal and middle segment respectively, of a right coronary artery (RCA), with significant tortuosity. (b)** Several slit-like stenotic lesions resembling accordion effect developed after successful wiring using a 0.014-inch Hi-torque Cross-IT guide-wire and balloon dilatation at the straightened segments (arrows). Multiple intracoronary administrations of nitrate did not resolve the newly appeared lesions.

**Figure 2:**
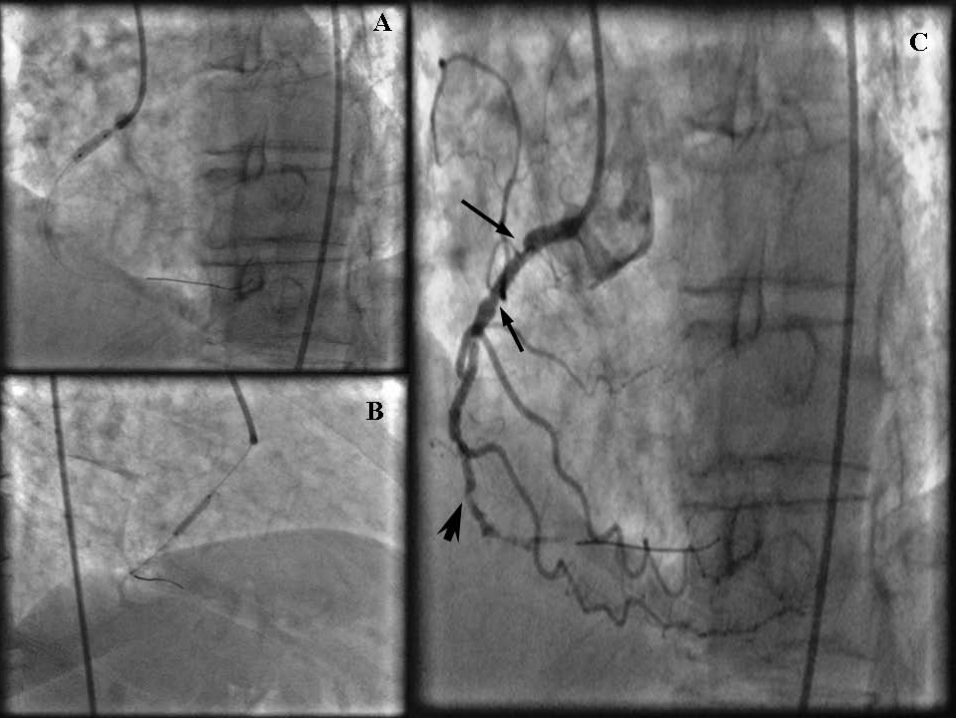
**(a,b) Stent deployment at the middle and proximal segment lesions due to persistent ST-segment elevation with hemodynamic instability.(c)** New stenosis at the proximal and distal edge of the proximal stent (arrows) and the distal edge of the peripheral lesion delivered stent (arrowhead). Despite intracoronary injection of isosorbide dinitrate the residual stenoses still remained, indicating a repeated accordion phenomenon.

**Figure 3:**
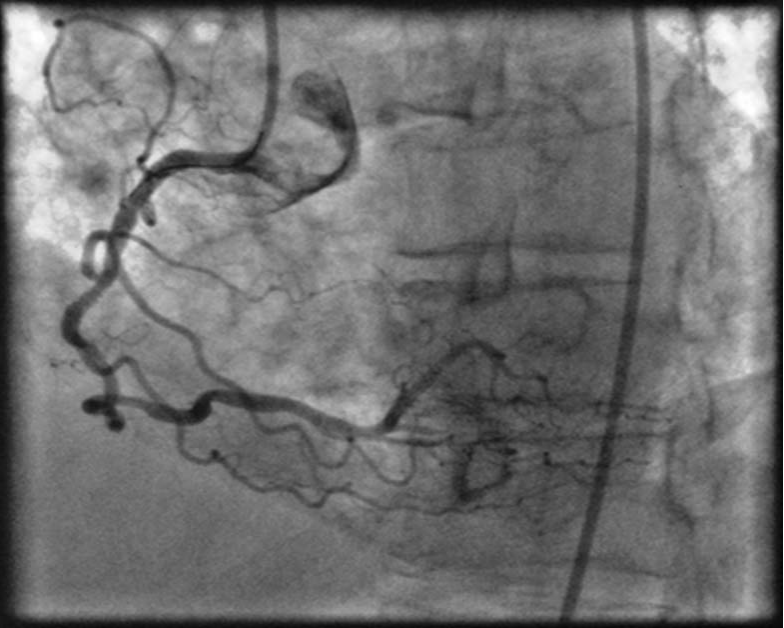
**Final coronary angiogram after removing the guidewire showing the disappearance of focal stenoses at both stents edges. **The restoration of proximal and mid segmental tortuosity resolved completely the newly developed lesions.

A non-elective diagnostic coronary angiogram (CAG) was performed with standard 6 F 4 Judkins (Cordis, Johnson&Johnson, Miami, FL, USA) catheters via a right femoral approach. Contrast injection revealed two stenotic lesions of 70% and 80%, at the proximal and middle segment respectively, of a right coronary artery (RCA), with significant tortuosity (Figure 1a). Coronary angioplasty of the right coronary artery was decided. After an unsuccessful attempt with a 0.014-inch Hi-torque Extra Suport (Guidant Corporation; Santa Clara, USA) guidewire to cross the lesions, a 0.014-inch Hi-torque Cross-IT (Guidant Corporation; Santa Clara, CA, USA), intermediate guidewire was advanced across the stenoses and balloon dilatation was performed with a 2 × 10 mm balloon (Flash, Bolton Medical, USA) at 12 atm. The tortuous proximal segments were completely straightened by this technique and after the balloon was removed, angiographic control exposed slit like multiple filling defects along the longitudinal axis of the RCA, an appearance of stenoses strongly suggesting the accordion effect or dissection, and vasospasm (Figure 1b). Isosorbide dinitrate was administrated intracoronary in order to clarify the possibility of coronary spasm but the angiographic appearance of the RCA did not change. The patient had severe chest pain with ECG changes (ST elevation on leads II, III, aVF) and a drop of blood pressure at critical levels (90/60 mmHg). As the deformity of the artery persisted and in view of patient's symptoms stenting was performed. A 2.75 mm × 14 mm stent (Apollo Cordynamic, Bionert, Barcelona, Spain) was delivered with little resistance at the middle segment lesion expanded at 16 atm. A second 3 mm × 14 mm stent (Apollo, Cordynamic, Bionert, Barcelona, Spain) was deployed at the proximal lesion which was expanded at 18 Atm (Figure 2a and 2b). Angiography following stenting showed residual stenosis at the edges of both stents (Figure 2c). Although coronary spasm was highly suspected, several intracoronary administrations of isosorbide dinitrate could not relieve of this narrowing. The appearance of newly developed lesion was thought as a repeated accordion phenomenon. Thus, the guidewire was withdrawn and this resulted in resolution of the new stenosis and resumption of previous proximal tortuosity of the artery (Figure 3). The final outcome was excellent. The patient had an uneventful recovery and was discharged after 24 hours.

## Discussion

Appearance of the accordion phenomenon, during routine angioplasty procedure, is not uncommon and is produced by mechanical alteration of the geometry and the curvature of the vessel due to straightening effect and shortening of the artery, preceded from guidewire or catheter balloon manipulation [1]. The highest incidence of accordion effect is seen when highly tortuous arterial vessels are linearized with a stiff guidewire and it can be simply be reversed by the withdrawn of the mechanical device causing the artery deformation [2].

The accordion phenomenon has been primarily reported in the era of PCI [1,3]. The right coronary artery is thought to be predominantly prone to this phenomenon because the artery is entrenched in the epicardial fat tissue and courses rather freely in the atrioventricular groove. It has also been rarely described in the internal mammary artery [4], the left main coronary artery [1], during percutaneous transluminal angioplasty of the iliac artery [5] and through carotid stenting [2].

A stiff guidewire is occasionally used to straighten the tortuous coronary arteries in order to achieve better accessibility to the distal target lesion and to avoid stent displacement from the balloon [6]. The coronary artery elongation however may induce angiographic defects-"web-like" eccentric constrictions-attributed to accordion phenomenon [7]. The latter can be inappropriately identified as coronary spasm, dissection or thrombus development, which may falsely lead to unnecessary stenting at the pseudo-narrowing lesion, turning a totally reversible event into a true iatrogenic complication [6]. Vasospasm is responsive to intracoronary vasodilators like nitroglycerin (100–200 μg) or calcium channel blockers. On the contrary it is well documented that vasodilators are ineffective in relieving pseudolesions and the only therapeutic management is to remove the angioplasty guide wire [8]. Intravascular ultrasound imaging may be helpful in this occasion to rule out dissection or thrombus existence prior to guide-wire removal [9].

Angioplasty with stenting on tortuous coronary arteries considered being difficult, due to hard stent delivery and possible displacement from proper position [8]. Even though, we encountered only a little resistance through stent deployment. Post-dilatation provided two new stenoses at edges of both stents. The recognition of the pre-stenting effect correctly certified the operators that this was a reversible phenomenon. Stiff guide wire withdrawal, facilitated the artery to restore its normal shape, with consequent disappearance of the pseudolesions and anginal symptoms.

## Conclusion

Even if accordion phenomenon is predictable in tortuous coronary arteries, in the presence of stenotic lesions, it can cause reversible narrowing and transient transmural myocardial ischemia. It is essential for interventional cardiologists to identify such iatrogenic events because they are basically benign and should be managed plainly by pulling outing the guidewire and re-establishing coronary geometry.

## Competing interests

The authors declare that they have no competing interests.

## Authors' contributions

GG conceived the case report. GG and LKP were involved in the case management, and drafted the manuscript. PA and AS performed the percutaneous coronary intervention and reviewed the draft manuscript and suggested revisions. GI and IA reviewed the manuscript and made the final corrections before submission. All the authors have read and approved the final manuscript.

## Consent

Written informed consent was obtained from the patient for publication of this case report and any accompanying images. A copy of the written consent is available for review by the Editor in-Chief of this journal.
